# Climate Vulnerability and Cardiometabolic Health Among Children

**DOI:** 10.1001/jamanetworkopen.2026.15205

**Published:** 2026-05-27

**Authors:** Eun Kyung Lee, Megan B. Cole

**Affiliations:** 1 Department of Environmental Studies, State University of New York, College of Environmental Science and Forestry, Syracuse; 2 Department of Population Medicine, Harvard Medical School & Harvard Pilgrim Health Care Institute, Boston, Massachusetts

## Abstract

This cross-sectional study evaluates the association of Climate Vulnerability Index quartiles with outpatient care and emergency department utilization for cardiometabolic diseases among pediatric patients in New York State.

## Introduction

Climate change is a growing public health crisis that disproportionately affects socioeconomically marginalized populations, highlighting environmental justice concerns regarding unequal exposure, protection, and policy involvement.^1^ Children are especially vulnerable, accounting for more than 88% of the global disease burden from climate hazards (eg, kidney, respiratory, and infectious diseases) due to their biological sensitivity and dependence on caregivers, health care, and community infrastructure. Cardiometabolic diseases (CMDs), including obesity, diabetes, and cardiovascular disease, are rising among US children. Weather-related events may be factors in increased CMD risk, particularly among low-income children disproportionately exposed to climate hazards.^2^ We examined CMD-related pediatric health care visits across various levels of climate vulnerability, stratified by insurance type.

## Methods

Our primary data source was the 2018 to 2022 New York State Department of Health Statewide Planning and Research Cooperative System, a comprehensive all-payer reporting system (eMethods in Supplement 1).^3^ We included data for all children aged 0 to 17 years residing in New York State. The Syracuse University Institutional Review Board approved this cross-sectional study and waived the informed consent requirement because deidentified data were used. We followed the STROBE reporting guideline.

The outcome included CMD-related outpatient and emergency department (ED) visits, with the primary CMD diagnoses identified using the first (principal) *International Statistical Classification of Diseases and Related Health Problems, Tenth Revision* code for each visit. The unit of analysis was visit-year.

Neighborhood climate vulnerability was assessed using the Climate Vulnerability Index (CVI),^4^ which incorporates baseline socioeconomic vulnerability and exposure to climate-related hazards at the Census-tract level. Neighborhoods were categorized into CVI quartiles (quartile 1 indicating lowest CVI; quartile 4 indicating highest CVI) based on patient residence (eFigure in Supplement 1).

We used Poisson regression models to estimate the association between CVI quartile and CMD-related health care visits, adjusting for age, sex, and self-reported or guardian-reported race and ethnicity. Analyses were stratified by insurance type (private, public, self-pay or no insurance), which served as a proxy for socioeconomic status. Results are presented as rate ratios (RRs) with 95% CIs. Analyses were conducted from September to December 2025 using SAS 9.4 (SAS Institute Inc).

## Results

The sample included 299 034 CMD-related health care visits (153 499 visits [51.3%] made by males; mean [SD] age, 7.5 [5.9] years) (Table 1). Overall, 38.2% of these visits were made by patients identifying as Hispanic, 4.0% as non-Hispanic Asian, 17.2% as non-Hispanic Black, 12.8% as non-Hispanic White, and 6.7% as other race and ethnicity. Most encounters were outpatient (86.3%), while 13.7% were ED visits. Across CVI quartiles, obesity accounted for 69.2% of visits, increasing from 55.7% in quartile 1 (lowest CVI) to 77.5% in quartile 4 (highest CVI).

**Table 1.** Pediatric Health Care Visit Characteristics Across Climate Vulnerability Index Quartiles in New York State, 2018-2022

Characteristic	CMD-related pediatric visits, No. (%)					*P* value^a^
	Total	Climate Vulnerability Index				
		Quartile 1 (lowest)	Quartile 2	Quartile 3	Quartile 4 (highest)	
No. of visits	299 034	74 258	74 795	75 321	74 660	
Age, mean (SD), y	7.5 (5.9)	7.5 (5.9)	7.5 (5.9)	7.5 (5.9)	7.5 (5.9)	<.001
Visit type						
ED	41 096 (13.7)	11 997 (16.2)	10 076 (13.5)	9451 (12.6)	9572 (12.8)	<.001
Outpatient	257 938 (86.3)	62 261 (83.8)	64 719 (86.5)	65 870 (87.5)	65 088 (87.2)	
Disease subtype						
CVD^b^	6107 (2.0)	2244 (3.0)	1475 (1.9)	1331 (1.8)	1057 (1.4)	<.001
Comorbidity^c^	2164 (0.72)	539 (0.73)	539 (0.72)	509 (0.68)	577 (0.77)	
Hyperlipidemia	15 389 (5.2)	5228 (7.0)	3939 (5.3)	3419 (4.5)	2803 (3.8)	
Hypertension	22 096 (7.4)	8342 (11.2)	5423 (7.3)	4607 (6.1)	3724 (4.9)	
Obesity	206 896 (69.2)	41 360 (55.7)	52 406 (70.1)	55 285 (73.4)	57 845 (77.5)	
T1D	29 743 (9.9)	12 010 (16.2)	6904 (9.2)	6131 (8.1)	4698 (6.3)	
T2D	16 639 (5.6)	4535 (6.1)	4109 (5.5)	4039 (5.4)	3956 (5.3)	
Race and ethnicity^d^						
Asian, non-Hispanic	11 960 (4.0)	3870 (5.2)	4214 (5.6)	3022 (4.0)	854 (1.1)	<.001
Black, non-Hispanic	51 294 (17.2)	8672 (11.7)	11 674 (15.6)	14 111 (18.7)	16 837 (22.6)	
Hispanic, any race	11 4194 (38.2)	19 117 (25.7)	28 845 (38.6)	32 413 (43.0)	33 819 (45.3)	
White, non-Hispanic	38 406 (12.8)	22 913 (30.9)	9353 (12.5)	4619 (6.1)	1521 (2.0)	
Other, non-Hispanic^e^	20 153 (6.7)	5580 (7.5)	5484 (7.3)	5232 (6.9)	3857 (5.2)	
Unknown^e^	63 027 (21.1)	14 106 (19.0)	15 225 (20.4)	15 924 (21.1)	17 772 (23.8)	
Sex						
Female	145 487 (48.7)	36 107 (48.6)	36 535 (48.9)	36 546 (48.5)	36 299 (48.6)	.91
Male	153 499 (51.3)	38 138 (51.4)	38 247 (51.1)	38 764 (51.5)	38 350 (51.4)	
Health insurance						
Private	163 263 (54.6)	37 150 (50.0)	42 905 (57.4)	40 438 (53.7)	42 770 (57.3)	<.001
Public^f^	89 495 (29.9)	24 390 (32.8)	23 149 (31.0)	22 127 (29.4)	19 829 (26.6)	
Self-pay or no insurance	11 806 (3.9)	3690 (5.0)	3139 (4.2)	2798 (3.7)	2179 (2.9)	
Other or unknown^f^	34 470 (11.5)	9028 (12.2)	5602 (7.5)	9958 (13.2)	9882 (13.2)	

Abbreviations: CMD, cardiometabolic disease; CVD, cardiovascular disease; ED, emergency department; T1D, type 1 diabetes; T2D, type 2 diabetes.

^a^ *P* values were 2-tailed, with statistical significance set at *P* < .05.

^b^ CVD included acute myocardial infarction, atrial fibrillation, congestive heart failure, stroke or transient ischemic attack, and ischemic heart disease.

^c^ Children with 2 or more types of CMDs.

^d^ Race and ethnicity were self-reported or guardian-reported. These data were included to describe visit characteristics and to adjust for race and ethnicity in Poisson models, accounting for potential differences by race and ethnicity.

^e^ Other race included non-Hispanic American Indian or Alaska Native, Native Hawaiian or Other Pacific Islander, and other. Unknown included all races classified without ethnicity.

^f^ Public insurance included Medicaid and Child Health Plus. Other insurance included coverage beyond private and public sources, such as federal employees program, automobile medical insurance, other nonfederal programs, and dental maintenance organizations.

Compared with quartile 1, quartile 4 had ED visit rates that were similarly elevated across insurance groups, with adjusted RRs (ARRs) of 3.62 (95% CI, 2.70-4.84) for patients with private insurance, 3.39 (95% CI, 2.86-4.01) for patients with public insurance, and 3.09 (95% CI, 2.16-4.42) for patients with self-pay or no insurance (Table 2). In contrast, outpatient visit increases in quartile 4 vs quartile 1 varied by insurance type, with the largest increase among children with public insurance (ARR, 5.31; 95% CI, 4.92-5.73), followed by children with self-pay or no insurance (ARR, 4.50; 95% CI, 3.50-5.78) and private insurance (ARR, 1.87; 95% CI, 1.67-2.09).

**Table 2.** Differential Associations of Climate Vulnerability With Cardiometabolic Health of Pediatric Patients by Health Insurance Status

Climate Vulnerability Index	Pediatric patients, RR (95% CI)					
	Private insurance		Public insurance		Self-pay or no insurance	
	Unadjusted	Adjusted^a^	Unadjusted	Adjusted^a^	Unadjusted	Adjusted^a^
**ED visits**						
Quartile 1 (lowest)	1 [Reference]	1 [Reference]	1 [Reference]	1 [Reference]	1 [Reference]	1 [Reference]
Quartile 2	1.61 (1.54-1.68)	1.35 (1.24-1.46)	1.12 (1.07-1.17)	1.19 (1.09-1.29)	1.03 (0.93-1.14)	1.28 (1.07-1.54)
Quartile 3	1.46 (1.40-1.53)	1.55 (1.38-1.74)	1.15 (1.09-1.20)	1.42 (1.28-1.58)	1.08 (0.97-1.20)	1.48 (1.20-1.83)
Quartile 4 (highest)	1.70 (1.63-1.77)	3.62 (2.70-4.84)	1.09 (1.04-1.15)	3.39 (2.86-4.01)	0.78 (0.69-0.88)	3.09 (2.16-4.42)
**Outpatient visits**						
Quartile 1 (lowest)	1 [Reference]	1 [Reference]	1 [Reference]	1 [Reference]	1 [Reference]	1 [Reference]
Quartile 2	1.89 (1.86-1.92)	1.07 (1.03-1.11)	1.23 (1.20-1.26)	1.34 (1.28-1.40)	1.11 (1.05-1.18)	1.60 (1.41-1.81)
Quartile 3	2.08 (2.04-2.11)	1.03 (0.98-1.09)	1.45 (1.42-1.48)	1.53 (1.45-1.62)	0.94 (0.88-1.00)	1.46 (1.25-1.71)
Quartile 4 (highest)	2.55 (2.51-2.60)	1.87 (1.67-2.09)	1.50 (1.46-1.53)	5.31 (4.92-5.73)	0.72 (0.67-0.77)	4.50 (3.50-5.78)

Abbreviations: ED, emergency department; RR, rate ratio.

^a^ Model adjusted for age, sex, and race and ethnicity.

## Discussion

In this all-payer cross-sectional study, higher neighborhood climate vulnerability was associated with increased CMD-related pediatric health care utilization, consistent with prior findings on adults.^5^ Contrary to existing literature reporting an association between private insurance and better access to outpatient care,^6^ we found that ED visits were highest among privately insured children, while outpatient visits predominated among publicly insured and self-pay or uninsured children in the most climate-vulnerable neighborhoods. This unexpected pattern may reflect differences in acute care utilization among privately insured children residing in areas with high CVI as well as pandemic-related changes in care delivery, including increased reliance on telehealth.

Study limitations include use of administrative data that mainly capture severe or billed cases; lack of individual-level clinical measures, which may underestimate conditions; limited temporal resolution of climate vulnerability exposure, including during the COVID-19 pandemic; and inability to account for duration of residence or repeat visits. Additionally, neighborhood-level CVI measurement may introduce ecological bias. Nonetheless, increased climate vulnerability—partly associated with changes in inflammatory markers, insulin sensitivity, indirect psychological stress, and limited access to social and health care systems—underscores the need for accessible care, resilient infrastructure, and targeted interventions in pediatric patients.

## Notes

Supplement 1. **eMethods.** New York State Department of Health (NYSDOH) Statewide Planning and Research Cooperative System (SPARCS) Data **eFigure.** Climate Vulnerability Index Scores in Quartiles Across New York State
eReferences

Supplement 2.
Data Sharing Statement
