# Supplementary material for: Climate Vulnerability and Cardiometabolic Health Among Children
Source: JAMA Netw Open. Author manuscript; Available in PMC 2026 May 28. (PMC13216978; doi:10.1001/jamanetworkopen.2026.15205)
Supplement: Supplementary Material 2 [file NIHMS2170597-supplement-Supplementary_Material_2.pdf]

Data Sharing Statement

Lee. Climate Vulnerability and Cardiometabolic Health Among Children. *JAMA Netw Open*. Published May 27, 2026. doi:10.1001/jamanetworkopen.2026.15205

Data
Data available: No

Additional Information
Explanation for why data not available: Due to patient confidentiality and in accordance with the data use agreement with the New York State Department of Health (NYSDOH), the data used for analysis will not be shared.
