# Supplementary material for: Climate Vulnerability and Cardiometabolic Health Among Children
Source: JAMA Netw Open. Author manuscript; Available in PMC 2026 May 28. (PMC13216978; doi:10.1001/jamanetworkopen.2026.15205)
Supplement: Supplementary Material 1 [file NIHMS2170597-supplement-Supplementary_Material_1.pdf]

Supplemental Online Content

Lee EK, Cole MB. Climate vulnerability and cardiometabolic health among children. *JAMA Netw Open*. 2026;9(5):e2615205. doi:10.1001/jamanetworkopen.2026.15205

eMethods. New York State Department of Health (NYSDOH) Statewide Planning and Research Cooperative System (SPARCS) Data

eFigure. Climate Vulnerability Index Scores in Quartiles Across New York State

eReferences

This supplemental material has been provided by the authors to give readers additional information about their work.

© 2026 Lee EK et al. *JAMA Network Open*

eMethods. New York State Department of Health (NYSDOH) Statewide Planning and Research Cooperative System (SPARCS) data

SPARCS data is a statewide, all-payer dataset established in 1979 as a result of cooperation between the healthcare industry and the government. SPARCS collects patient-level information on demographics, diagnoses and treatments, services, and charges for each hospital visit. By law, New York State hospitals are required to report encounter-level data within 60 days of discharge, and historical compliance has been approximately 95% statewide. SPARCS data undergo automated validation checks at submission, including required field verification, logical consistency checks, and confirmation of standardized ICD-10 and CPT coding. Facilities must correct any records that fail validation prior to acceptance, and NYSDOH conducts ongoing data quality monitoring.

For more information on SPARCS data: https://www.health.ny.gov/statistics/sparcs/

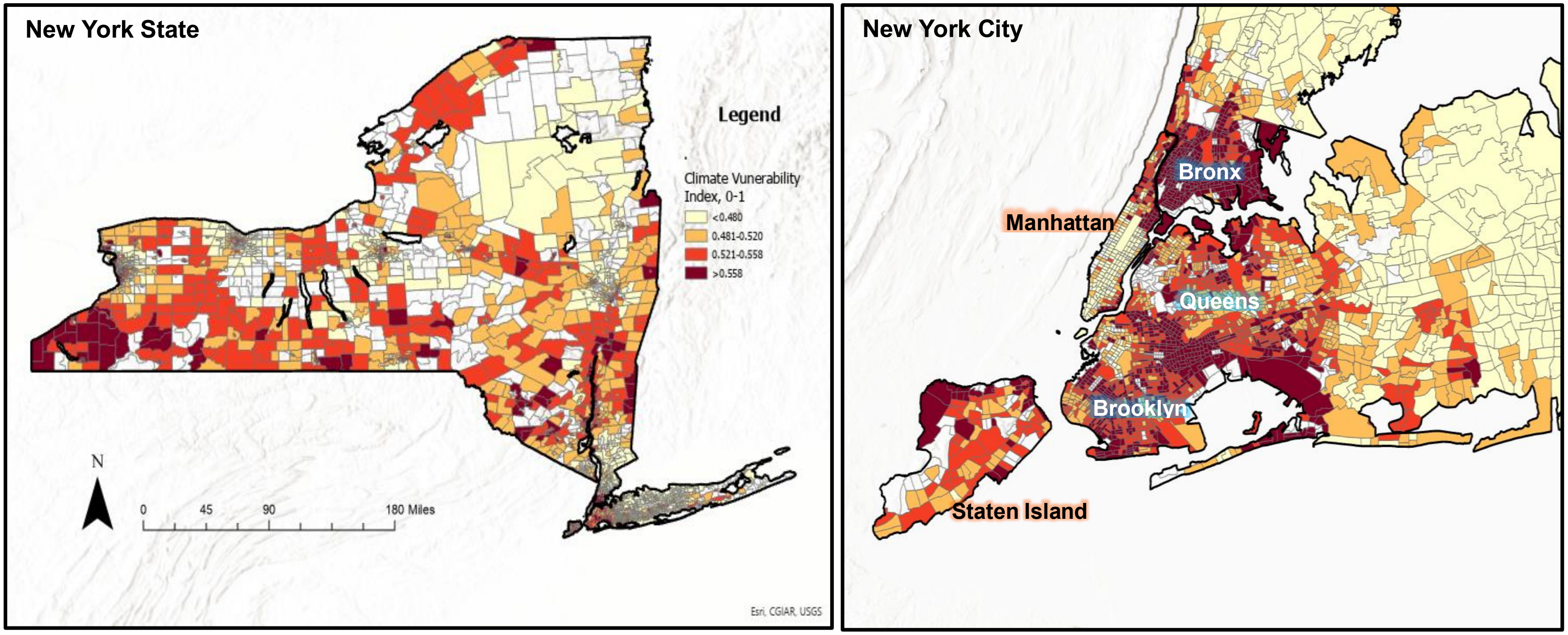

eFigure. Climate vulnerability index scores in quartiles across New York State.

Quartiles cut points: Q1:<0.480; Q2: 0.481-0.520; Q3: 0.521-0.558; Q4: >0.558

The Climate Vulnerability Index (CVI), an environmental justice screening and mapping tool, was developed by the Environmental Defense Fund and Texas A&M University.[1] The CVI assesses community vulnerabilities and climate-related risks. It is constructed at the census tract level and includes more than 200 national census tracts. The index incorporates 184 indicators across seven domains, organized into two main themes: (1) baseline vulnerabilities: baseline health, socioeconomic conditions, infrastructure, and environment; and (2) climate change risks: climate change-related health impacts, climate change-related socioeconomic conditions (e.g., damage, loss, and costs), and extreme events.[2]

© 2026 Lee EK et al. *JAMA Network Open*

eReferences

1. Environmental Defense Fund, Texas A&M University. The U.S. Climate Vulnerability Index. 2023. Accessed January 30, 2025. https://climatevulnerabilityindex.org/methodology/

2. Tee Lewis PG, Chiu WA, Nasser E, et al. Characterizing vulnerabilities to climate change across the United States. *Environ Int*. 2023;172. doi:10.1016/j.envint.2023.107772

© 2026 Lee EK et al. *JAMA Network Open*
